# FACTORS ASSOCIATED WITH THE OUTCOMES OF OLDER PATIENTS OPERATED DUE TO HIP FRACTURES

**DOI:** 10.1590/1413-785220233102e259371

**Published:** 2023-05-01

**Authors:** FERNANDO GONZALEZ CORRÊA, LUAN TOSHIO SERIKAWA, ROBERTO BEZERRA NICOLAU, LUIS FELIPE BRANDT FERRES, JOÃO CARLOS PEDRO FILHO, FERNANDO BALDY DOS REIS, LUIZ FERNANDO COCCO

**Affiliations:** 1 Universidade Federal de Sao Paulo, Escola Paulista de Medicina, Departamento de Ortopedia e Traumatologia, Sao Paulo, SP, Brazil.

**Keywords:** Hip Fractures, Femoral Fractures, Mortality, Retrospective Studies, Risk Factors, Orthopedic Procedures, Fraturas do Quadril, Fraturas do Fêmur, Mortalidade, Estudos Retrospectivos, Fatores de Risco, Procedimentos Ortopédicos

## Abstract

**Objective:**

Evaluating clinical factors associated with mortality in older patients who underwent surgical correction of hip fractures.

**Methods:**

This observational and retrospective study analyzed the medical records of 67 patients (aged older than 60 years), both men and women, who underwent surgical correction of hip fractures from 2019 to 2020 at Hospital São Paulo. The following variables were analyzed: age, sex, presence of comorbidities, affected hip region, and trauma mechanism. Statistical analyses were performed using the SPSS software.

**Results:**

The mean age of patients was 78.12 ± 9.80 years and 80.6% of the sample were women. The prevalence of hip fractures on the right side (52.2%), in the transtrochanteric region (53.7%), and due to fall on the same level (88.1%) was higher. Systemic arterial hypertension (77.6%), diabetes mellitus (37.3%), and dementia (16.4%) were frequent comorbidities. The prevalence of death after fracture was 17.9% and it was associated with longer hospital stay after surgery (p = 0.028).

**Conclusion:**

The prevalence of mortality of patients with hip fractures who underwent surgery was 17.9%. A longer hospital stay due to pre-existing comorbidities was the main factor related to this outcome. ***Level of Evidence III, Retrospective Study.***

## RESUMO

**Objetivo:**

Avaliar os fatores clínicos associados à mortalidade em pacientes idosos submetidos ao tratamento cirúrgico para correção de fraturas do quadril.

**Métodos:**

Estudo observacional e retrospectivo de análise de prontuários de 67 pacientes (idade superior a 60 anos). Foram incluídos indivíduos de ambos os sexos, submetidos ao tratamento cirúrgico para correção de fraturas do quadril, entre 2019 e 2020, no Hospital São Paulo. Foram analisadas as variáveis: idade, sexo, presença de comorbidades, região do quadril acometido e mecanismo de trauma. As análises estatísticas foram realizadas pelo software SPSS.

**Resultados:**

A idade média dos participantes foi de 78,12 ± 9,80 anos, e 80,6% da amostra era constituída por mulheres. Houve maior prevalência de fraturas do quadril no lado direito (52,2%), na região transtrocantérica (53,7%) e causada por queda sem desnível (88,1%). Hipertensão arterial sistêmica (77,6%), diabetes mellitus (37,3%) e demência (16,4%) foram frequentes. A prevalência de óbito após a fratura foi de 17,9%, sendo associada a maior tempo de internação hospitalar após a cirurgia (p = 0,028).

**Conclusão:**

A prevalência de mortalidade dos pacientes com fratura do quadril submetidos à cirurgia foi de 17,9%. O tempo prolongando de internação hospitalar por comorbidades pré-existentes foi o principal fator relacionado a este desfecho. **Nível de Evidência III, Estudo Retrospectivo.**

## INTRODUCTION

Population aging is a worldwide phenomenon in a progressive increase. By 2050, the number of older adults in the world is estimated to be about 2 billion.^1^ The epidemiology of fractures also tends to increase, with 4.5 million cases estimated for 2050. ^(^ ^2^ In Brazil, population aging poses new challenges for health services, since, as the older population increases, fragility fractures (hip, shoulder, wrist, and spine) affect an increasingly significant number of individuals. ^(^ ^3^ ^)-(^ ^7^

In general, treatment in geriatric trauma needs special attention, as it requires specialized services and longer hospital stay, increasing morbidity and mortality and the costs of health services. ^(^ ^3^ ^), (^ ^8^ In this scenario, hip fractures stand out, since they are associated with severe complications, such as increased rates of admission in intensive care units, risk of infection, and mortality. ^(^ ^9^ ^)-(^ ^11^

High-energy mechanisms such as traffic accidents, falls from great heights, and firearm injuries are the most frequent cause of fractures among young adults, whereas low-energy traumas such as simple falls are more frequent among older adults with less unstable fracture traits. ^(^ ^9^ ^), (^ ^12^ Moreover, fracture characteristics, anatomical site, and severity can also be influenced by factors related to bone density quality. ^(^ ^12^ ^), (^ ^13^

We found other peculiarities in the process of fracture healing in older adults. A decrease in the healing potential results in lower rates of healing or bone nonunion and, consequently, higher mortality risk. ^(^ ^3^ ^), (^ ^12^

Among factors associated with mortality after hip fracture, advanced age, female sex, the presence of comorbidities, surgical delay, the type of anesthesia, and altered laboratory tests play an important role. ^(^ ^8^ ^), (^ ^9^ Thus, handling with manageable risks in the treatment schedule of older patients with proximal femoral fractures is mandatory. ^(^ ^13^ Moreover, studies on the outcomes of these patients are essential for the development of national policies focused on assisting health care professionals in creating more effective preventive strategies.

Thus, this study aims to evaluate the clinical factors related to mortality in older patients who underwent surgical correction of hip fractures at Hospital São Paulo.

## METHODS

### Type of study

This observational and retrospective study collected data from the medical records of patients treated at Hospital São Paulo by the Department of Orthopedics and Traumatology of the Paulista School of Medicine (UNIFESP). The study respects the ethical and legal aspects of studies with human beings and was approved by the Research Ethics Committee of the Universidade Federal São Paulo (no. 5.143.826).

### Casuistry

This study was based on the analysis of medical records of patients older than 60 years, both men and women, who underwent surgical correction of hip fractures at Hospital São Paulo from January 1, 2019, to January 1, 2020, with outpatient follow-up for one year. Patients with incomplete or outdated medical records or who did not agree with the terms of consent to participate in the study were excluded. Deaths during hospitalization or outpatient follow-up were considered.

### Data collection

Researchers analyzed cases exclusively by electronic medical records. The following data were collected: age, sex, presence of comorbidities (systemic arterial hypertension and diabetes mellitus), trauma mechanism, type of fracture (intracapsular or extracapsular), preoperative time (from admission to surgery), postoperative time (from surgery to hospital discharge), total length of hospital stay, type of surgery (osteosynthesis or arthroplasty), clinical outcome (survival or mortality), laterality, and autonomy to walk before fracture. Researchers neither collected data in person nor had access to the name or hospital record of patients, ensuring their anonymity.

### Statistical analysis

Data were placed in an Excel spreadsheet for further analysis regarding age, sex, and pre-existing diseases related to clinical outcomes after surgical treatment of fractures. Descriptive analyses were presented in absolute numbers (n) and relative frequencies (%), along with mean and standard deviation. Statistical analyses were performed using the SPSS software (version 21). For statistical significance, the established cutoff value was p < 0.05.

## RESULTS

This study included 67 older patients, with a mean age of 78.12 ± 9.80 years. Most patients were women (80.6%; n = 54). Among comorbidities at admission, systemic arterial hypertension (77.6%; n = 52) stood out, followed by diabetes mellitus (37.3%; n = 25). Older patients with some degree of dementia confirmed by geriatric clinical examinations represented 16.4% (n = 11) of the sample. Most cases were femoral fractures on the right side (52.2%; n = 35) and transtrochanteric fractures (53.7%; n = 36). Among patients, 64.2% (n = 43) walked almost exclusively at home and 34.3% (n = 23) were able to walk both inside and outside the home before fracture. The main cause of femoral fracture was fall from the same level (88.1%; n = 59). The prevalence of death due to femoral fracture was 17.9% (n = 12) even after surgery. Regarding hospital parameters, length of hospital stay was 8.45 ± 5.63 days, preoperative time was 3.32 ± 3.91 days, and postoperative time was 4.52 ± 3.894 days (Table 1).

**Table 1:** Characteristics of older patients who underwent surgical correction of hip fractures at Hospital São Paulo from 2016 to 2021.

Variables	Frequency	%
**Gender**		
Men	13	19.4
Women	54	80.6
Total	67	100
**Systemic arterial hypertension**		
No	15	22.4
Yes	52	77.6
Total	67	100
**Diabetes mellitus**		
No	42	62.7
Yes	25	37.3
Total	67	100
**Dementia**		
No	56	83.6
Yes	11	16.4
Total	67	100
**Laterality**		
Right	35	52.2
Left	32	47.8
Total	67	100
**Functional status**		
Walked at home	43	64.2
Walked inside and outside home	23	34.3
Other^#^	1	1.5
Total	67	100
**Diagnosis**		
Femoral neck fracture	23	34.3
Transtrochanteric fracture	36	53.7
Other^%^	8	11.9
**Cause**		
Fall from the same level	59	88.1
Traffic accident	1	1.5
Other*	7	10.4
Total	67	100
**Final outcome**		
Non-mortality	55	82.1
Mortality	12	17.9
Total	67	100

^#^Bedridden, and paraplegic patients and wheelchair users; ^%^Multiple femoral fractures and diaphyseal femoral fractures; *Unwitnessed fall, prophylactic fixation (myeloma), physical aggression, and pathologic fracture; n: sample number.

To analyze epidemiological and clinical factors that influenced outcomes, patients were divided into two groups: non-mortality (82.10 %; n = 55) and mortality (17.90%; n = 12). However, groups had no significant difference between them (Table 2)

**Table 2:** Association of epidemiological and clinical aspects with the outcome in patients who underwent surgical correction of hip fractures.

Variables	Non-mortality (n = 55)		Mortality (n = 12)		p
	n	%	n	%	
**Gender**					0.227^#^
Men	9	16.40%	4	33.30%	
Women	46	83.60%	8	66.70%	
**SAH**					0.443^#^
No	11	20.00%	4	33.30%	
Yes	44	80.00%	8	66.70%	
**DM**					0.751^*^
No	35	63.60%	7	58.30%	
Yes	20	36.40%	5	41.70%	
**Dementia**					0.400^#^
No	47	85.50%	9	75.00%	
Yes	8	14.50%	3	25.00%	
**Laterality**					1.000^*^
Right	29	52.70%	6	50.00%	
Left	26	47.30%	6	50.00%	
**Diagnosis**					0.449^#^
Femoral neck fracture	17	30.90%	6	50.00%	
Transtrochanteric fracture	31	56.40%	5	41.70%	
Other^%^	7	12.70%	1	8.30%	
**Functional status**					0.743^*^
Walked at home	36	65.50%	7	58.30%	
Other^&^	19	34.50%	5	41.70%	
**Cause**					1.000^#^
Fall from the same level	48	87.30%	11	91.70%	
Other“	7	12.70%	1	8.30%	

^#^Fisher’s exact test; *Chi-square test; ^%^Multiple femoral fractures and diaphyseal femoral fractures; ^&^Bedridden and paraplegic patients and wheelchair users; “Unwitnessed fall, prophylactic fixation (myeloma), physical aggression, and pathologic fracture; p < 0.05: statistical significance; n: sample number; SAH: systemic arterial hypertension; DM: diabetes mellitus.

Regarding numerical variables, patients with mortality outcome had longer hospital stay (11.67 ± 7.19 days) compared to patients with non-mortality outcome (7.75 ± 5.045 days) (p = 0.028; Table 3). Patients who died also had higher mean age, more comorbidities, and longer preoperative and postoperative time. However, we found no significant differences (p ≥ 0.050).

**Table 3:** Association of age, number of comorbidities, length of hospital stay, preoperative and postoperative time with clinical outcomes of patients who underwent surgical correction of hip fractures.

	Non-mortality (n = 55)		Mortality (n = 12)		p
Variables	Mean	SD	Mean	SD	
**Age**	77.870	10.123	79.250	8.487	0.663
**Number of comorbidities**	2.150	1.183	2.330	0.985	0.620
**Length of hospital stay (days)**	7.750	5.045	11.670	7.190	**0.028**
**Preoperative time (days)**	4.360	3.602	5.250	5.154	0.581
**Postoperative time (days)**	3.190	2.727	3.920	7.366	0.592

^#^Student’s t-test; n: sample number; SD: standard deviation; p < 0.05: statistical significance.

To assess if length of hospital stay increased mortality risk, a binary regression model was made, including the variables length of hospital stay and preoperative and postoperative time. Hospitalization time increases the risk of death with borderline p-values (OR = 1.127; p = 0.057) (Table 4).

**Table 4:** Binary logistic regression of the influence of length of hospital stay on the clinical outcome of patients who underwent surgical correction of hip fractures.

		95%CI		
Variables	OR	Lower	Upper	p
**Length of hospital stay**	1.127	0.997	1.275	0.057
**Preoperative time**	1.017	0.855	1.210	0.850
**Postoperative time**	0.959	0.804	1.142	0.636

OR: odds ratio; CI: confidence interval; p < 0.05: statistical significance.

## DISCUSSION

This study aimed to retrospectively evaluate factors associated with mortality after surgical correction of hip fractures in older adults. The prevalence of mortality after surgery was 17.9%, which was associated with longer hospital stay due to pre-existing comorbidities.

Hip fractures represent a growing public health problem worldwide and are associated with higher mortality in older patients. ^(^ ^3^ Among hip fractures, proximal femoral fractures stood out, especially transtrochanteric fractures due to their high frequency among older adults and significant social and economic impact. ^(^ ^14^ Our study is in line with other studies that show the prevalence of intertrochanteric fractures in relation to the femoral neck. ^(^ ^8^ ^),(^ ^10^ Moreover, our sample has a profile similar to the literature, considering the occurrence in older and women, with comorbidities such as diabetes mellitus and arterial hypertension. ^(^ ^3^ ^), (^ ^8^ ^)-(^ ^10^ ^), (^ ^13^ A study with 38,126 women with hip fractures and a six-year follow-up time showed that the presence of comorbidities before fracture was associated with high short-term mortality and reduced survival in 39% of cases. Thus, due to the increasing number of patients with comorbidities associated with aging and high mortality in patients with hip fractures, improving preoperative and postoperative care for patients with chronic heart, kidney, or lung diseases can potentially reduce mortality. ^(^ ^15^

Brazil has a high prevalence of hip fractures and high mortality and morbidity rates associated with this condition. ^(^ ^3^ ^), (^ ^8^ ^), (^ ^10^ In this study, hip fracture mortality rates were similar to previous studies. ^(^ ^16^ ^)-(^ ^18^

In a study conducted in Brazil, the mortality rate was 14.4% among patients with femoral fractures and this mortality was associated with increasing age, leukocytosis, need for intensive care, and no surgical treatment. Correa et al. ^(^ ^10^ showed that the mortality rate among older adults with proximal femoral fractures at the national level was 8% and male sex, reduced Parker score, delirium diagnosed at hospital admission or developed during hospitalization, and surgical delay were the main associated risk factors.

In the United States, a study presented a femoral fracture mortality rate of 1.6%.^11^ The authors concluded that patients have a high risk of mortality and postoperative complications if they undergo surgical fixation 48 hours after admission, with longer ICU and hospital stay and increased need for mechanical ventilation. ^(^ ^11^

As one of the main causes of hospitalization of older patients, hip fractures require careful perioperative management to avoid complications and decrease mortality rates. Most of the available guidelines recommend surgical correction of fractures in the first 24 hours, up to 48 hours, as an increased waiting time is correlated with higher medium-term complication and mortality rates. Moreover, diagnostic-therapeutic care proved to significantly reduce the length of hospital stay. ^(^ ^19^

Similarly to our findings, studies show that a longer hospital stay negatively affects older patients with femoral fractures, as it contributes to the development of other hospital complications such as infection, pressure ulcers, and sepsis, besides increasing mortality rate. ^(^ ^8^ ^), (^ ^9^ ^), (^ ^18^ According to a recent demographic study, the southeastern region of Brazil showed the highest femoral fracture mortality rates and the longest total hospital stay. ^(^ ^3^ Thus, a longer hospital stay may contribute to higher mortality rates in this region, which is in line with our findings.

With population aging, optimizing length of hospital stay and reducing overload in health and social services is essential. ^(^ ^3^ Mortality risk may be high, especially due to the low bone quality of older patients. ^(^ ^3^ ^), (^ ^12^ ^), (^ ^13^ Thus, preventive measures to reduce fractures among this population are extremely important.

Despite the limitations of this study due to its retrospective nature, development in a single center, and small sample size, we could identify the association between hip fracture mortality in older adults and length of hospital stay. Our data can be used in the planning of services and care protocols focused on older patients with femoral fractures in order to reduce mortality risk. This should occur within a multidisciplinary approach, including hospital care and improvements in the availability of rehabilitation services. However, further epidemiological studies with a larger sample size are necessary to analyze the association of hip fracture mortality in older adults, since studies on this topic are scarce.

## CONCLUSION

In this study, the prevalence of mortality of patients with hip fractures who underwent surgery was 17.9%, and a longer hospital stay due to comorbidities was the main factor related to this outcome.
